# Artificial Intelligence Anxiety and Patient Safety Attitudes Among Operating Room Professionals: A Descriptive Cross-Sectional Study †

**DOI:** 10.3390/healthcare13162021

**Published:** 2025-08-16

**Authors:** Pinar Ongun, Burcak Sahin Koze, Yasemin Altinbas

**Affiliations:** 1 Department of Surgical Nursing, Faculty of Health Sciences, Balıkesir University, Balikesir 10010, Türkiye; 2 Department of Surgical Nursing, Faculty of Nursing, Ege University, Izmir 35100, Türkiye; burcak.sahin.koze@ege.edu.tr; 3 Department of Surgical Nursing, Faculty of Health Sciences, Adıyaman University, Adıyaman 02030, Türkiye; yaltinbas@adiyaman.edu.tr

**Keywords:** artificial intelligence, patient safety, operating room, healthcare professional attitudes

## Abstract

Background/Objectives: The adoption of artificial intelligence (AI) in healthcare, particularly in high-stakes environments such as operating rooms (ORs), is expanding rapidly. While AI has the potential to enhance patient safety and clinical efficiency, it may also trigger anxiety among healthcare professionals due to uncertainties around job displacement, ethical concerns, and system reliability. This study aimed to examine the relationship between AI-related anxiety and patient safety attitudes among OR professionals. Methods: A descriptive, cross-sectional research design was employed. The sample included 155 OR professionals from a university and a city hospital in Turkey. Data were collected using a demographic questionnaire, the Artificial Intelligence Anxiety Scale (AIAS), and the Safety Attitudes Questionnaire–Operating Room version (SAQ-OR). Statistical analyses included t-tests, ANOVA, Pearson correlation, and multiple regression. Results: The mean AIAS score was 3.25 ± 0.8, and the mean SAQ score was 43.2 ± 10.5. Higher AI anxiety was reported by males and those with postgraduate education. Participants who believed AI could improve patient safety scored significantly higher on AIAS subscales related to learning, job change, and AI configuration. No significant correlation was found between AI anxiety and safety attitudes (r = −0.064, *p* > 0.05). Conclusions: Although no direct association was found between AI anxiety and patient safety attitudes, belief in AI’s potential was linked to greater openness to change. These findings suggest a need for targeted training and policy support to promote safe and confident AI adoption in surgical practice.

## 1. Introduction

Patient safety includes risk assessment, management, and identification of patient-related risks, as well as reporting and analysis to reduce recurrent risks and effectively implement solutions [1]. Globally, patient safety remains a critical issue, as highlighted in the Global Patient Safety Action Plan 2021–2030. It is a leading cause of death and disability worldwide, underscoring the need for risk mitigation strategies. Increased use of AI technologies has been recommended to reduce human error and improve patient safety [2].

AI refers to the ability of algorithms within technological systems to learn from data and perform automated tasks without requiring explicit programming for each step [1]. In healthcare, AI has the potential to provide clinicians with enhanced resources, improve patient care, ensure accurate diagnoses, and optimize treatment plans [1]. A variety of smart health applications are being developed for these purposes, including AI-powered robots, mobile applications, wearable sensors, and smart devices [3,4,5].

Operating rooms are inherently complex environments where interdisciplinary teams work together with patient safety at the forefront. Research shows that AI technologies can significantly improve surgical safety by reducing complications and preventing unnecessary surgical deaths. For example, autonomous robotic systems have outperformed expert surgeons in performing precise tasks such as intestinal anastomosis [6]. Similarly, sensor-based AI technologies have improved surgeons’ ability to delineate tumor boundaries, enabling safer and more effective surgeries [7]. In addition, innovations such as robotic operating room nurses and instrument-cleaning robots within AI-integrated systems have demonstrated improved efficiency and effectiveness in maintaining sterile surgical environments [8].

Despite the promising potential of AI to improve patient safety, there remains a significant gap in understanding healthcare professionals’ attitudes toward both patient safety and AI technologies. While advances in AI have sparked debates about the potential for AI to replace human roles in healthcare [3], limited research has examined how these developments are perceived by healthcare providers, particularly in high-risk settings such as operating rooms. A study conducted by Filiz (2022) in Turkey assessed the levels of anxiety toward artificial intelligence among physicians, nurses, and health technicians using the Artificial Intelligence Anxiety Scale. Drawing on data collected from 330 healthcare professionals, the findings indicated that nurses exhibited the highest levels of AI-related anxiety, whereas physicians reported the lowest [9].

The existing literature highlights the need for research that focuses on healthcare professionals’ perceptions, concerns, and attitudes toward AI in the context of patient safety. In particular, there is a lack of studies that examine the perceptions of operating room professionals regarding the integration of AI technologies and their potential impact on patient safety practices. To address this gap, the present study aims to explore the relationship between OR professionals’ attitudes toward patient safety and their fears of AI. In doing so, the study seeks to contribute to a deeper understanding of how emerging technologies can be effectively and safely integrated into healthcare environments.

## 2. Materials and Methods

### 2.1. Type of Study and Research Questions

This study uses a descriptive, cross-sectional research design. The research questions addressed in this study are as follows:

I. What is the level of artificial intelligence anxiety among operating room professionals?

II. What is the mean safety attitude score of operating room professionals?

III. Is there a relationship between artificial anxiety and operating room professionals’ safety attitudes?

IV. What factors influence AI anxiety and the safety attitudes of operating room professionals?

### 2.2. Study Setting and Time

The study was conducted in the operating rooms of a university hospital and a city hospital in Turkey between 22 February 2023 and 19 June 2023.

### 2.3. Population and Sampling

The study population consisted of nurses, physicians, and anesthesia technicians working in the operating rooms of a University Health Practice and Research Hospital and a City Hospital. A total of 155 participants, including nurses, physicians, and anesthesia technicians who consented to participate, formed the study sample. Non-probability sampling was used. Healthcare workers on long-term medical leave, such as pregnancy leave, postpartum leave, or companion leave, were excluded from the study. The rationale for this exclusion was that these individuals were not actively engaged in clinical duties during the data collection period, and their absence from the work environment could significantly affect the variables of interest. To ensure adequate familiarity with the organizational culture and workflow, a minimum of one month was considered sufficient for inclusion. This criterion is consistent with similar studies in the literature, which required participants to work for at least three weeks [10] and up to one year [11].

### 2.4. Data Collection

Data were collected through face-to-face interviews using a “data collection form” designed by the researchers based on a review of the literature, along with the “Artificial Intelligence Anxiety Scale” and the “Safety Attitudes Questionnaire—Operating Room Version”. The Artificial Intelligence Anxiety Scale (AIAS) and the Safety Attitudes Questionnaire–Operating Room version (SAQ-OR) were chosen not only because they are validated and reliable instruments in Turkish but also because they are directly aligned with the aims of this study. The AIAS measures individuals’ concerns and perceptions regarding AI technologies, which is central to assessing AI-related anxiety among operating room professionals. The SAQ-OR, on the other hand, specifically evaluates safety attitudes within surgical environments, making it an appropriate tool for exploring perceptions of patient safety in the operating room context. The high internal consistency values (0.93 for AIAS and 0.92 for SAQ-OR) further support their suitability for the present study.

Data Collection Form: The data collection form included questions addressing demographic and professional characteristics such as age, gender, marital status, educational level, occupation, years of professional experience, and years working in the operating room. Additional questions focused on knowledge and perceptions of AI, including awareness of AI applications in surgery, devices employing AI technology in surgical contexts, the use of AI for enhancing patient safety in the operating room, the role of AI in surgical procedures, and potential ethical challenges posed by AI technology [11,12,13].

Artificial Intelligence Anxiety Scale (AIAS): The Artificial Intelligence Anxiety Scale (AIAS) was developed by Wang and Wang (2019) [14] as a tool to assess participants’ anxiety regarding their current AI experience. It employs a 5-point Likert scale ranging from not at all (1 point) to completely (5 points). The scale comprises 16 items divided into four subscales: Learning (items 1–5), Job Change (items 6–9), Socio-technical Blindness (items 10–13), and AI Configuration (items 14–16). Scores range from 16 to 80 (Table 1), with higher scores indicating greater levels of AI anxiety. The Turkish adaptation, including validity and reliability testing, was conducted by Akkaya (2021) [15], who reported an internal consistency coefficient of 0.93 for the scale. In the current study, the internal consistency coefficient was also calculated as 0.93, confirming its reliability. The mean total score on the AIAS was 3.2 ± 0.8. Sub-dimension mean scores were as follows: Learning (3.6 ± 0.9), Job Change (3.2 ± 1.0), Socio-technical Blindness (2.8 ± 0.9), and AI Configuration (3.1 ± 1.1) (Table 1).

Safety Attitude Questionnaire–OR Version: The Safety Attitudes Scale (SAQ) was developed by Sexton et al. (2006) at the University of Texas to assess attitudes toward patient safety [13]. The operating room version of the scale was adapted into Turkish by Onler (2019) [11]. The scale consists of a total of 58 items grouped into five subscales. These include team cooperation, job satisfaction, perception of management, safe environment, working conditions, and determination of stress level. It should be noted that some items of the scale (1, 12, 16, 24, 25, 27, 31, 32, 33, 36, 39, 44, 47, 49, 52, 53, 56, 58) contain negative statements and are reverse-coded. Scale scores are converted according to the following scheme: 1 = 0, 2 = 25, 3 = 50, 4 = 75, and 5 = 100. The lowest possible score is “0”, while the highest possible score is “100”. The scale has no predetermined cut-off point. As the score increases, so does the level of safety attitudes. The total internal consistency coefficient was calculated to be 0.92. In this study, the internal consistency coefficient was calculated as 0.88 (Table 1).

The mean total score for the SAQ scale was 43.2 ± 10.5. Further analysis of the mean scores according to the subscales of the scale revealed the following: the mean score of the team cooperation subscale was 35.9 ± 10.8, the mean score of the job satisfaction subscale was 35.6 ± 19.7, and the mean score of the management thoughts subscale was 45.9. The mean score of the safe environment subscale was 43.4 ± 10.6, the mean score of the working conditions subscale was 42.4 ± 20.3, and the mean score of the stress level subscale was 35.9 ± 13.2 (Table 1).

### 2.5. Data Analysis

Data were analyzed using the SPSS 25 statistical software package. Descriptive statistics, including numbers, means, percentage distributions, and standard deviations, were calculated. The Kolmogorov–Smirnov Z-test was used to assess the normality of the data distribution. Since the test did not reject the normality hypothesis, parametric tests were used. A t-test was used to compare quantitative continuous data between two independent groups, while a one-way ANOVA test was used for comparisons between more than two independent groups. Tukey’s post hoc analysis was used to further analyze differences identified by the ANOVA test. Pearson correlation analysis was used to examine relationships between two continuous variables. Multiple regression analyses were performed to identify factors influencing the results. A *p*-value of less than 0.05 was considered statistically significant for all analyses.

### 2.6. Ethical Considerations

The study was approved by the Balıkesir University Non-Interventional Scientific Research Ethics Committee (Number: 2022/110 on 6 December 2022). All participating healthcare professionals were informed about the study, both verbally and in writing. Consent was also obtained from the authors who developed and validated the scales used in the study. The study was conducted by the tenets of the Declaration of Helsinki.

## 3. Results

The average age of the OR professionals who participated in the study was 35.7 *±* 7.9 years. Among them, 50.3% were male, and 68.4% were married. The mean length of professional experience was 150.4 ± 99.4 months, with an average of 81.8 ± 75.7 months spent in the operating room. In addition, 58.7% (n = 91) of the participants had completed postgraduate education. Regarding workplace distribution, 51.0% (n = 79) of the professionals worked in a university hospital, while 70.3% (n = 109) were unfamiliar with the specific applications of AI in surgery. Among the AI technologies used in surgical applications, robotic surgery was the most recognized, with 94.8% (n = 147) of participants reporting awareness. In addition, 65.2% (n = 101) of participants believed that AI technology could improve patient safety in the operating room, and 62.6% (n = 97) expressed that AI technology would not lead to ethical concerns (Table 2).

A chi-square test was conducted to examine the association between the participants’ responses to the questions “Can Artificial Intelligence Technology Be Used to Ensure Patient Safety in the Operating Room?” and “Does the Use of Artificial Intelligence Technology in Surgical Applications Cause Ethical Problems?” The analysis revealed no statistically significant relationship between the two variables (χ^2^ = 2.789, *p* = 0.095) (Table 3).

Males had significantly higher mean scores than females on the learning subscale (t = −3.791, *p* < 0.001, 95% CI: −0.862 to −0.271), the AI configuration subscale (t = −2.416, *p* = 0.017, 95% CI: −0.785 to −0.078), and the total AI Anxiety Scale score (t = −2.925, *p* = 0.004, 95% CI: −0.622 to −0.120). Participants with postgraduate education had higher mean scores than those with other levels of education on the AI Anxiety Scale total score (F = 3.651, *p* = 0.014, 95% CI: 3.232 to 3.584), the learning subscale (F = 3.000, *p* = 0.032, 95% CI: 3.642 to 4.058), and the AI configuration subscale (F = 4.099, *p* = 0.008, 95% CI: 3.149 to 3.648). These differences were statistically significant. In addition, participants who were aware of AI applications scored significantly higher on the AI configuration subscale than those who were not (t = 2.008, *p* = 0.046, 95% CI: 0.006 to 0.784). Participants who believed that AI technology could improve patient safety in the operating room had significantly higher scores on the learning subscale (t = 3.198, *p* = 0.002, 95% CI: 0.194 to 0.822), the job change subscale (t = 2.383, *p* = 0.018, 95% CI: 0.073 to 0.786), the AI configuration subscale (t = 2.842, *p* = 0.005, 95% CI: 0.161 to 0.897), and the total AI anxiety scale score (t = 3.315, *p* = 0.001, 95% CI: 0.177 to 0.700), compared to those who did not share this belief. Conversely, those who believed that AI technology in surgical applications would lead to ethical problems had significantly higher scores on the job change subscale (t = −4.170, *p* < 0.001, 95% CI: −1.053 to −0.376), the socio-technical blindness subscale (t = −4. 904, *p* < 0.001, 95% CI: −1.025 to −0.436), the AI configuration subscale (t = −4.939, *p* < 0.001, 95% CI: −1.209 to −0.518), and the total AI anxiety scale score (t = −4.879, *p* < 0.001, 95% CI: −0.860 to −0.364) compared to those who did not perceive ethical concerns (Table 4).

The analysis revealed that single participants scored significantly higher on the job satisfaction subscale than married participants (t = −2.214, *p* = 0.028, 95% CI: −14.115 to −0.803) (Table 5). Participants with an associate’s degree had higher mean scores on the thoughts about management sub-dimension (F = 4.151, *p* = 0.007, 95% CI: 42.581 to 51.452), the safe environment subscale (F = 3.266, *p* = 0.023, 95% CI: 41.804 to 46.269), and the SAQ total score (F = 3.114, *p* = 0.028, 95% CI: 41.880 to 46.524) compared to participants with other levels of education (Table 5). Participants with postgraduate education scored significantly higher on the working conditions subscale of the SAQ (F = 11.290, *p* < 0.001, 95% CI: 44.075 to 52.628) compared to participants with other levels of education (Table 5).

When comparing the total and subscale scores of the SAQ and the Artificial Intelligence Anxiety Scale (AIAS), no significant relationship was found between the two scales or their respective subscales (r = −0.064, *p* = 0.429).

Multiple regression analysis was used to identify factors that influence safety attitudes and fear of AI. In the multiple regression analysis, independent variables were selected based on the results of prior univariate analyses. Only variables with a significance level of *p* < 0.05 in these preliminary tests were included in the regression models. To analyze the determinants of safety attitudes, the independent variables included educational status, occupation, and AI anxiety (Table 6). The results showed that the regression equation did not exhibit multicollinearity problems based on the variance inflation factor (VIF) values. The adjusted R2 of the independent variables together explained 6% of the variance in safety attitudes. The overall model was statistically significant (F = 4.537, *p* = 0.004).

A similar regression analysis was conducted to determine the factors influencing AI anxiety. Independent variables for this model included gender, perception of whether AI technology can ensure patient safety, and safety attitudes (Table 6). The results showed that the regression equation did not exhibit multicollinearity problems based on the variance inflation factor (VIF) values. The adjusted R^2^ of the independent variables accounted for 8% of the variation in AI anxiety. The model was statistically significant (F = 5.567, *p* = 0.001).

## 4. Discussion

Artificial intelligence technologies are increasingly being integrated into healthcare to protect patient health and improve treatment outcomes. AI contributes to early and accurate diagnosis, treatment, patient care, and advances in healthcare research. However, the implementation of AI in healthcare has raised critical discussions about patient safety, privacy, and the ethical implications of AI-driven systems, becoming a focal topic for healthcare experts and society at large [1,2]. International organizations such as the WHO and the OECD have initiated guidelines and policies to address these concerns by examining whether healthcare professionals are adequately informed about the increasing applications of AI in their field [1]. This study aims to explore the relationship between patient safety attitudes and AI-related anxiety among healthcare professionals working in the operating room.

### 4.1. Artificial Intelligence Technologies and Healthcare Professionals

Studies examining healthcare professionals’ perspectives on AI show that many are only beginning to familiarize themselves with technology. As a result, they often lack clarity about its potential impact, leading to concerns about how AI could positively or negatively impact their profession [16]. In one study, AI anxiety scores varied significantly among different healthcare professionals, with physicians scoring lower (60.26) than nurses (71.67) and technicians (69.46) [9]. The researchers attributed this disparity to physicians being more knowledgeable about AI and its applications in their fields. In contrast, other healthcare professionals showed higher levels of anxiety, fearing that AI could potentially replace their profession [9]. The results of this study suggest that operating room professionals generally experience moderate levels of AI-related anxiety. This is consistent with the broader observation that AI has not yet been fully integrated into the healthcare sector. Of the participants, 70.30% reported being unfamiliar with AI applications in surgical practice, and overall familiarity was low. While AI technologies have the potential to free up physicians’ time for patient interactions, they also raise concerns about professional responsibilities. Physicians may feel the need to improve their skills in communicating risks, discussing treatment options, and demonstrating their competence in AI systems [1]. The moderate levels of anxiety observed among healthcare professionals may reflect their ongoing process of understanding and adapting to the innovations and changes brought about by AI. These findings underscore the importance of providing comprehensive training and support to healthcare professionals to facilitate the acceptance and effective implementation of AI in clinical settings. In addition to general training, targeted AI literacy programs, particularly those involving real-case simulations and hands-on digital skills workshops, may further enhance confidence and engagement among professionals.

### 4.2. Artificial Intelligence Technologies and Patient Safety

In healthcare institutions, AI is designed to streamline hospital workflows and improve patient satisfaction. A 2023 executive order outlines specific safety goals for AI, emphasizing the need to define frameworks for identifying AI-related clinical errors, analyzing data, developing guidelines for improvement, and effectively communicating those guidelines to healthcare providers. This approach highlights the potential for the controllability of AI to play a critical role in improving patient safety [17]. While some healthcare professionals recognize the positive contributions of AI, others express concern about its potential risks to patient safety [18]. A primary concern is the risk of compromising patient privacy. The ease of access to sensitive patient information raises fears about the security of personal data. In addition, ethical dilemmas may arise, particularly regarding the potential misuse of data and the impact of AI on clinical decision making [19,20].

One study examined how various AI techniques could improve patient safety by addressing major adverse events associated with healthcare, including infections, adverse drug events, venous thromboembolism, surgical complications, pressure ulcers, falls, and diagnostic errors. Research highlights the critical role of AI-enabled sensors, wearable technologies, and electronic health records in the early detection, prediction, and prevention of these events [21]. A separate study found that AI will be more systematic and less emotional than humans, reducing the likelihood of errors [18]. Even though 65.2% of our participants indicated that AI technology could be used to ensure patient safety in the operating room, it was found that their level of anxiety about AI was relatively high. It is imperative that OR professionals demonstrate an understanding and belief that this technology can be used to improve patient safety, despite their elevated level of anxiety about AI. This is considered an essential step in the acceptance and integration of AI-based solutions in healthcare. Successful integration, however, requires more than individual belief; it depends heavily on organizational readiness. Leadership commitment, allocation of resources, and the cultivation of a culture that embraces innovation are all critical factors that shape whether AI technologies are accepted, trusted, and effectively utilized in clinical settings.

### 4.3. Artificial Intelligence Technologies and Ethical Issues

The integration of digital technologies into the operating room can significantly improve surgical workflow. With the development of electronic data systems, healthcare professionals can access patient information at any time. However, this convenience raises ethical concerns, particularly about patient privacy [20]. In addition, changes in AI coding could potentially harm patients, which raises ethical objections [19]. As AI is an advanced technology, access restrictions may be imposed, which, in turn, could undermine the principle of equality in healthcare [22]. A study on the ethical use of AI found consensus on principles such as justice, non-maleficence, and responsibility, while other principles such as privacy, solidarity, human dignity, and sustainability were less emphasized [23]. In our study, we observed that participants who believed that AI technology would not lead to ethical issues in surgical applications (62.6%) had a higher mean total score on the AIAS than those who believed that it would lead to ethical issues. This finding contrasts with the results of most other studies, which suggest that the use of AI may lead to ethical challenges. The different results in our study may reflect the growing prevalence of AI technology and its perceived need to ensure patient safety. AI, which is expected to meet certain objectives with the principle of do no harm in order to serve humanity and is becoming widespread day by day, is thought to increase the AIA levels of OR professionals in terms of application, operation, problem management, and continuity due to the expectation that it will be built on an environmentally friendly, reliable, and ethical form, as well as the unknowns in the process. In conclusion, based on the existing literature, discussions about the ethical implications of AI in healthcare will continue, especially given the limited implementation of AI technologies in the field [1].

### 4.4. AIAS Mean Scores

The results of the AIAS (Table 4) show that the mean subscale scores for males were higher than those for females. Previous studies have shown that individuals with higher levels of education tend to have more positive attitudes toward AI technology and lower levels of anxiety [24,25]. In this study, the higher mean total score on the AIAS among participants with postgraduate education compared to those with other levels of education may reflect concerns about AI potentially replacing the jobs of healthcare professionals. In addition, while 147 participants were familiar with robotic devices used in AI technology, only 49 were familiar with the Cyberknife device. As AI technology and these devices become more widely adopted in the future, healthcare professionals may become more knowledgeable and familiar with their use.

### 4.5. Mean SAQ Scores

The results of the SAQ in this study showed that the mean total score for males was higher than that for females. However, another study found that the mean total score for females was higher than that for males [11]. In a study by Sogut (2019) that included operating room professionals, job satisfaction received the highest score, while the safety environment received the lowest score [26]. Based on these findings, it is likely that the professional responsibilities of operating room professionals contribute to increased stress levels. Additionally, single individuals had a higher mean total score (45.16) than married individuals, and those with an associate degree had the highest mean total score compared to participants with other education levels. In contrast, another study found that individuals with postgraduate education had the lowest mean total score [11].

### 4.6. Relationship Between AIAS and SAQ

Participants who were familiar with AI had higher mean total scores on both the AIAS and the SAQ. Those who agreed that AI technology could be used to ensure patient safety in the operating room also had higher mean total scores. Interestingly, those who believed that AI could cause ethical problems had a higher mean total score on the SAQ. It should be noted that the more complex technological advancements are, the more complex the ethical issues that arise. Considering that great power brings great responsibility, whether AI-enabled devices or care providers, touted as possessing great power, will harm people or other entities, and their responsibility for patient safety are important ethical debates. Therefore, AI’s ability to work with humans in a trustworthy manner is only possible if ethical AI, encompassing transparency, responsibility, confidentiality, and accountability, is built by OR professionals who have a high level of safety attitude. However, in this study, when the total and subscale mean scores of both the AIAS and SAQ were examined, no significant relationship was found between attitudes toward patient safety and AI anxiety levels among OR professionals. In the literature, there is a lack of studies that examine the relationship between AIAS and SAQ of OR professionals. In contrast, a study found a significant and negative correlation between nurses’ attitudes toward artificial intelligence and their AIA levels [27]. A separate study found that there was a statistically significant, weak, and negative correlation between the organizational stress scale and the SAQ of OR staff [28].

One key limitation of this study is its cross-sectional design, which captures participants’ attitudes and anxiety levels toward AI at a single point in time. Given that AI adoption is a dynamic and ongoing process, these perceptions may evolve as professionals gain more exposure and experience. Future longitudinal research is recommended to monitor such changes over time and provide deeper insights into the evolving nature of AI integration in healthcare.

Another limitation is that participants were recruited from only two hospitals in Turkey. Consequently, the findings may not be representative of operating room professionals working in other regions or within different healthcare systems, limiting the generalizability of the results.

## 5. Conclusions

This study found no statistically significant correlation between artificial intelligence anxiety and patient safety attitudes among operating room professionals. However, those familiar with AI applications demonstrated higher scores on the AI configuration subscale, and participants who believed in AI’s potential to enhance patient safety exhibited greater openness to learning, job-related changes, and AI integration. Conversely, individuals concerned about ethical implications reported higher levels of anxiety, particularly in domains related to job change and sociotechnical uncertainty. These findings suggest that attitudes toward AI are shaped not only by knowledge and belief in its benefits but also by perceived risks and ethical ambiguity. To support the safe and effective adoption of AI in surgical environments, healthcare institutions should prioritize targeted education, transparent communication, and structured strategies that build trust and competence in digital health technologies.

## Figures and Tables

**Table 1:** The total and subscale mean scores for the Artificial Intelligence Anxiety Scale and the Safety Attitude Questionnaire.

Scale	Sub-Dimensions	Mean ± SD	Items
AIAS	Learning	3.6 ± 0.9	1–5
	Job change	3.2 ± 1.0	6–9
	Sociotechnical blindness	2.1 ± 0.9	10–13
	AI configuration	3.1 ± 1.1	14–16
	Scale mean	3.2 ± 0.8	1–16
SAQ	Team collaboration	35.9 ± 10.8	3, 13, 14, 19, 24, 30, 34, 35, 37, 38, 39, 43, 50, 58
	Job satisfaction	35.6 ± 19.7	2, 8, 15, 29, 41
	Management considerations	45.9 ± 21.0	7, 9, 10, 17, 18, 22, 26
	Safe environment	43.4 ± 10.6	4, 5, 11, 12, 20, 21, 27, 28, 36, 44, 45, 46, 48, 51, 54, 56, 57
	Working conditions	42.4 ± 20.3	6, 23, 42
	Stress level	35.9 ± 13.2	1, 16, 25, 31, 32, 33, 40, 47, 49, 52, 53, 55
	Scale total score mean	43.2 ± 10.5	1–58

AIAS = Artificial Intelligence Anxiety Scale; SAQ = Safety Attitude Questionnaire; AI = Artificial Intelligence; SD = Standard Deviation.

**Table 2:** Distribution of sociodemographic characteristics of operating room professionals (n = 155).

Characteristics	Mean ± SD	
Age (year)	35.7 ± 7.9	
Total months of professional experience	150.5 ± 99.4	
Total months of experience in the operating room	81.9 ± 75.7	
**Gender**	**n**	**%**
Female	77	49.7
Male	78	50.3
**Marital Status**		
Married	106	68.4
Single	49	31.6
**Educational Status**		
Health Vocational High School	11	7.1
Associate degree	16	10.3
Bachelor’s degree	37	23.9
Postgraduate	91	58.7
**Occupation**		
Operating Room Nurse	37	23.8
Specialist doctor	37	23.9
Faculty Member	16	10.3
Assistant	33	21.3
Anesthesia technician	28	18.1
Other	4	2.6
**Hospital Worked in**		
City hospital	76	49.0
University Hospital	79	51.0
**Knowledge of the usage areas of AI in surgery**		
Yes	46	29.7
No	109	70.3
Previously heard about devices that enable the use of AI in surgical applications *		
Robotics	147	94.8
Cyberknife	49	31.6
Smart Knife	34	21.9
Wearable Technology	24	15.5
Decision Support Systems in Surgery	7	4.5
**AI technology can be used to ensure patient safety in the operating room.**		
Yes	101	65.2
No	54	34.8
**AI technology may lead to ethical issues in surgical applications.**		
Yes	58	37.4
No	97	62.6

SD = standard deviation; AI = artificial intelligence. * Participants made more than one mark.

**Table 3:** Crosstabulation of “Can Artificial Intelligence Technology Be Used to Ensure Patient Safety in the Operating Room?” and “Does the Use of Artificial Intelligence Technology in Surgical Applications Cause Ethical Problems?” (n = 155).

		AI Technology May Lead to Ethical Issues in Surgical Applications		Total
		Yes	No	
**AI technology can be used to ensure patient safety in the operating room**	**Yes**	33	68	101
	**No**	25	29	54
		X^2^ = 2.789 *p* = 0.095		
**Total**		58	97	155

X^2^ = chi-square test; AI = artificial intelligence; *p* < 0.05.

**Table 4:** Distribution and comparison of participants’ mean scores based on selected characteristics (N = 155).

Characteristics	Learning	Job Change	Sociotechnical Blindness	AI Configuration	AIAS Total
	Mean ± SD	Mean ± SD	Mean ± SD	Mean ± SD	Mean ± SD
**Gender**					
Female	3.4 ± 0.9	3.1 ± 1.0	2.7 ± 0.9	3.0 ± 1.0	3.1 ± 0.7
Male	4.0 ± 1.0	3.4 ± 1.1	2.9 ± 1.0	3.4 ± 1.2	3.4 ± 0.9
	t = −3.791 *p* < 0.001 **	t = −1.522 *p* = 0.130	t = −1.228 *p* = 0.221	t = −2.416 *p* = 0.017 *	t = −2.925 *p* = 0.004 *
**Marital Status**					
Married	3.6 ± 1.0	3.2 ± 1.1	2.8 ± 1.0	3.2 ± 1.2	3.2 ± 0.8
Single	3.7 ± 0.9	3.3 ± 1.0	2.9 ± 1.0	3.3 ± 1.1	3.3 ± 0.8
	t = −0.433 *p* = 0.666	t = −0.549 *p* = 0.584	t = −0.624 *p* = 0.534	t = −0.547 *p* = 0.585	t = −0.675 *p* = 0.501
**Educational Status**					
Health Vocational High School	3.6 ± 0.8	3.5 ± 0.2	3.0 ± 0.9	3.2 ± 0.7	3.3 ± 0.7
Associate degree	3.4 ± 1.0	3.2 ± 0.2	3.0 ± 0.9	3.2 ± 0.9	3.2 ± 0.7
Bachelor’s degree	3.4 ± 0.9	2.9 ± 0.2	2.5 ± 0.8	2.7 ± 1.0	2.9 ± 0.7
Postgraduate	3.9 ± 1.0	3.4 ± 0.1	2.9 ± 1.0	3.4 ± 1.2	3.4 ± 0.9
	F = 3.000 *p* = 0.032 *	F = 1.645 *p* = 0.181	F = 1.801 *p* = 0.150	F = 4.099 *p* = 0.008 *	F = 3.651 *p* = 0.014 *
**Awareness of** **AI** **Applications**					
Yes	3.8 ± 1.0	3.4 ± 1.0	2.9 ± 0.9	3.5 ± 1.1	3.4 ± 0.8
No	3.6 ± 1.0	3.2 ± 1.1	2.8 ± 1.0	3.1 ± 1.1	3.2 ± 0.8
	t = 1.130 *p* = 0.260	t = 0.932 *p* = 0.353	t = −0.987 *p* = 0.325	t = 2.008 *p* = 0.046 *	t = 1.556 *p* = 0.122
**Can** **AI** **Technology Be Used to Ensure Patient Safety in the Operating Room?**					
Yes	3.8 ± 1.0	3.4 ± 1.1	2.9 ± 1.0	3.4 ± 1.1	3.4 ± 0.8
No	3.3 ± 0.9	3.0 ± 1.0	2.6 ± 0.9	2.8 ± 1.0	3.0 ± 0.8
	t = 3.198 *p* = 0.002 *	t = 2.383 *p* = 0.018 *	t = 1.817 *p* = 0.071	t = 2.842 *p* = 0.005 *	t = 3.315 *p* = 0.001 *
**Does the Use of** **AI** **Technology in Surgical Applications Cause Ethical Problems?**					
Yes	3.5 ± 1.0	2.8 ± 1.0	2.3 ± 0.8	2.6 ± 1.1	2.9 ± 0.7
No	3.8 ± 1.0	3.5 ± 1.0	3.1 ± 0.9	3.5 ± 1.0	3.5 ± 0.8
	t = −1.781 *p* = 0.077	t = −4.170 *p* < 0.001 **	t = −4.904 *p* < 0.001 **	t = −4.939 *p* < 0.001 **	t = −4.879 *p* < 0.001 **

* *p* < 0.05; ** *p* < 0.001; t = independent T-test, F = ANOVA; SD = standard deviation; AI = artificial Intelligence.

**Table 5:** Distribution and comparison of SAQ mean scores based on participant characteristics (N = 155).

Characteristics	Team Collaboration	Job Satisfaction	Management Considerations	Safe Environment	Working Conditions	Stress Level	Total
	Mean ± SD	Mean ± SD	Mean ± SD	Mean ± SD	Mean ± SD	Mean ± SD	Mean ± SD
**Gender**							
Female	34.6 ± 9.3	35.7 ± 17.9	46.4 ± 19.0	44.4 ± 10.0	39.6 ± 17.5	36.5 ± 12.9	42.9 ± 9.6
Male	37.2 ± 12.2	35.5 ± 21.6	45.6 ± 23.0	42.6 ± 11.2	45.3 ± 22.6	35.4 ± 13.6	43.6 ± 11.4
	t = −1.522 *p* = 0.130	t = 0.063 *p* = 0.950	t = 0.257 *p* = 0.798	t = 1.050 *p* = 0.295	t = −1.751 *p* = 0.082	t = 0.484 *p* = 0.629	t = −0.406 *p* = 0.685
**Marital Status**							
Married	36.0 ± 11.6	33.3 ± 19.3	44.1 ± 20.7	42.6 ± 10.6	40.8 ± 20.7	36.2 ± 13.1	42.3 ± 10.6
Single	35.8 ± 9.3	40.7 ± 20.0	50.1 ± 21.5	45.5 ± 10.7	46.1 ± 19.3	35.4 ± 13.6	45.2 ± 10.2
	t = 0.113 *p* = 0.910	t = −2.214 *p* = 0.028 *	t = −1.652 *p* = 0.101	t = −1.612 *p* = 0.109	t = −1.509 *p* = 0.133	t = 0.371 *p* = 0.711	t = −1.570 *p* = 0.119
**Educational Status**							
Health Vocational High School	31.0 ± 7.8	24.6 ± 10.1	30.2 ± 24.6	38.2 ± 10.6	17.4 ± 10.8	42.8 ± 8.7	35.4 ± 8.3
Associate degree	37.3 ± 10.4	42.8 ± 23.0	57.4 ± 24.0	49.3 ± 8.3	41.7 ± 19.2	40.4 ± 12.1	46.4 ± 12.9
Bachelor’s degree	33.7 ± 8.3	33.5 ± 14.3	43.2 ± 14.4	41.2 ± 10.4	35.8 ± 14.4	35.1 ± 13.6	41.8 ± 6.8
Postgraduate	37.2 ± 12.0	36.5 ± 23.0	47.0 ± 21.3	44.0 ± 10.7	48.4 ± 20.5	34.7 ± 13.4	44.2 ± 11.2
	F = 1.804 *p* = 0.149	F = 2.109 *p* = 0.056	F = 4.151 *p* = 0.007 *	F = 3.266 *p* = 0.023 *	F = 11.290 *p* < 0.001 **	F = 1.941 *p* = 0.125	F = 3.114 *p* = 0.028 *
**Awareness of** **AI** **Applications**							
Yes	36.3 ± 11.1	35.3 ± 20.5	47.2 ± 20.4	44.2 ± 9.5	45.8 ± 19.6	34.0 ± 11.7	44.1 ± 10.9
No	35.8 ± 10.8	35.7 ± 19.5	45.5 ± 21.4	43.2 ± 11.1	41.1 ± 20.6	36.8 ± 13.8	42.8 ± 10.4
	t = 0.241 *p* = 0.810	t = −0.117 *p* = 0.907	t = 0.466 *p* = 0.642	t = 0.506 *p* = 0.613	t = 1.338 *p* = 0.183	t = −1.191 *p* = 0.236	t = 0.702 *p* = 0.484
**Can** **AI Technology Be Used to Ensure Patient Safety in the Operating Room?**							
Yes	37.2 ± 11.2	35.3 ± 21.2	46.8 ± 22.0	44.3 ± 10.8	44.2 ± 21.4	36.8 ± 13.6	43.3 ± 11.5
No	33.7 ± 10.0	36.1 ± 17.0	44.6 ± 19.2	42.1 ± 10.3	39.2 ± 18.0	34.3 ± 12.5	43.0 ± 8.5
	t = 1.196 *p* = 0.057	t = 2–0.245 *p* = 0.807	t = 0.611 *p* = 0.542	t = 1.229 *p* = 0.221	t = 1.470 *p* = 0.144	t = 1.143 *p* = 0.255	t = 0.183 *p* = 0.855
**Does the Use of** **AI** **Technology in Surgical Applications Cause Ethical Problems?**							
Yes	36.4 ± 11.4	37.9 ± 19.0	49.0 ± 19.6	43.9 ± 11.0	48.8 ± 18.9	33.4 ± 13.4	44.8 ± 9.5
No	35.7 ± 10.6	34.2 ± 20.1	44.2 ± 21.8	43.2 ± 10.4	41.7 ± 21.3	37.5 ± 12.9	42.3 ± 11.1
	t = 0.378 *p* = 0.706	t = 1.131 *p* = 0.260	t = 1.360 *p* = 0.176	t = 0.413 *p* = 0.680	t = 0.636 *p* = 0.525	t = −1.901 *p* = 0.059	t = 1.421 *p* = 0.157

* *p* < 0.05; t = independent T-test, ** *p* < 0.001; F = ANOVA; SD = standard deviation; AI = artificial intelligence.

**Table 6:** Regression analysis results for the SAQ and the AIAS.

Variable	β	t	*p*	VIF Values
	2.374	12.374	0.000	
Education Status	0.095	2.588	0.011	1.077
Occupation	0.056	3.053	0.003	1.064
AIAS	−0.042	−1.042	0.299	1.013
**Dependent Variable: Safety Attitudes** **Independent Variable: Education status, occupation, AIAS** F = 4.537, *p* = 0.004, R^2^ = 0.083, Adjusted R^2^ = 0.064. *p* < 0.05, Durbin Watson = 1.629				
**Variable**	**β**	**t**	***p***	**VIF values**
	3.703	7.315	0.001	
Gender	0.281	2.166	0.032	1.083
AI technology can be used to ensure patient safety in the OR	−0.359	−2.639	0.009	1.082
SAQ	−0.140	0.942	0.348	1.001
**Dependent Variable: Artificial Intelligence Anxiety** **Independent Variable: gender, AI technology can be used to ensure patient safety in the OR, Safety Attitudes** F = 5.567, *p* = 0.001, R^2^ = 0.100, Adjusted R^2^ = 0.082. *p* < 0.05, Durbin Watson = 1.836				

## Data Availability

Data are contained within the article. The original contributions presented in this study are included in the article. Further inquiries can be directed to the corresponding author.
